# Liver transplantation for decompensated cirrhosis of uncertain etiology in a woman with long-standing untreated panhypopituitarism: a case report

**DOI:** 10.3389/fendo.2026.1899455

**Published:** 2026-07-28

**Authors:** Lu Wang, Xiaojuan Ye, Wenwen Luo, Wei Zhang, Longliu Qian, Jiahuang Huang

**Affiliations:** 1 Department of General Practice, The First Affiliated Hospital of Shenzhen University, Shenzhen Second People’s Hospital, Shenzhen, Guangdong, China; 2 Department of Pathology, The Third Affiliated Hospital of Sun Yat-Sen University, Guangzhou, China; 3 Department of Gastroenterology, The First Affiliated Hospital of Shenzhen University, Shenzhen Second People’s Hospital, Shenzhen, Guangdong, China

**Keywords:** hypopituitarism, pituitary adenoma, GH/IGF-1 axis, MASLD, cirrhosis, liver transplantation, case report

## Abstract

**Background:**

Hypopituitarism is associated with adverse body composition and steatotic liver disease, particularly when the growth hormone/insulin-like growth factor-1 (GH/IGF-1) axis is disturbed. Progression to decompensated cirrhosis is uncommon, and published reports do not establish a direct causal relationship.

**Case presentation:**

A 37-year-old woman presented with jaundice, massive ascites, splenomegaly, thrombocytopenia, hypoalbuminemia, coagulopathy, and hyponatremia. She had undergone pituitary adenoma surgery at approximately 12 years of age, followed by amenorrhea and more than two decades without regular endocrine follow-up or hormone replacement. The clinical and biochemical profile supported long-standing panhypopituitarism. Possible central HPA-axis dysfunction was present; secondary adrenal insufficiency was clinically probable but not dynamically confirmed. Other features included central hypothyroidism, hypogonadotropic hypogonadism, low prolactin, central diabetes insipidus, and severe GH/IGF-1 axis disturbance. GH stimulation testing was not performed, and interpretation of the GH/IGF-1 axis was confounded by advanced liver disease. Common viral, autoimmune, alcohol-related, drug-related, and overt cardiac causes were not supported. However, transferrin saturation was 94.6%, and iron overload, Wilson disease, alpha-1 antitrypsin deficiency, and histological metabolic steatohepatitis were not fully excluded. Liver biopsy supported advanced fibrosis/cirrhosis, and explant pathology showed micronodular cirrhosis with hepatocellular cholestasis. She underwent liver transplantation in May 2026. Aminotransferase levels subsequently declined, but persistent thrombocytopenia, pulmonary infection, and acute kidney injury requiring hemodialysis developed. She progressed to septic shock and died more than 20 days after transplantation.

**Conclusion:**

Long-standing untreated panhypopituitarism may have contributed to metabolic and fibrogenic risk in this patient, but incomplete etiological evaluation precluded a definitive causal link. Lifelong endocrine follow-up and attention to liver health are warranted after pituitary surgery.

## Introduction

Hypopituitarism after pituitary or parasellar disease may affect the adrenal, thyroid, gonadal, GH/IGF-1, prolactin, and posterior pituitary axes (1). Beyond classical endocrine manifestations, chronic hormonal deficiencies can alter body composition, insulin sensitivity, hepatic lipid handling, inflammation, and fibrogenic signaling (2–4). Observational studies have reported a high burden of metabolic dysfunction-associated steatotic liver disease (MASLD) among adults with hypopituitarism or GH deficiency (5–7).

Progression to decompensated cirrhosis or liver transplantation is rare and has been described mainly in isolated reports involving childhood-onset hypothalamic-pituitary disease, steatohepatitis, or hepatopulmonary syndrome (8–14). We report an adult woman with long-standing untreated panhypopituitarism who required liver transplantation for decompensated cirrhosis of uncertain etiology. The case illustrates this unusual coexistence without establishing hypopituitarism as the sole cause.

## Case presentation

A 37-year-old woman presented with abdominal distension, followed by 10 days of watery diarrhea. She was 162 cm tall and weighed 73.6 kg (BMI, 28.04 kg/m²), although ascites and edema inflated this value. She reported no alcohol, smoking, medication or supplement exposure, or family history of liver disease.

**Figure 1:**
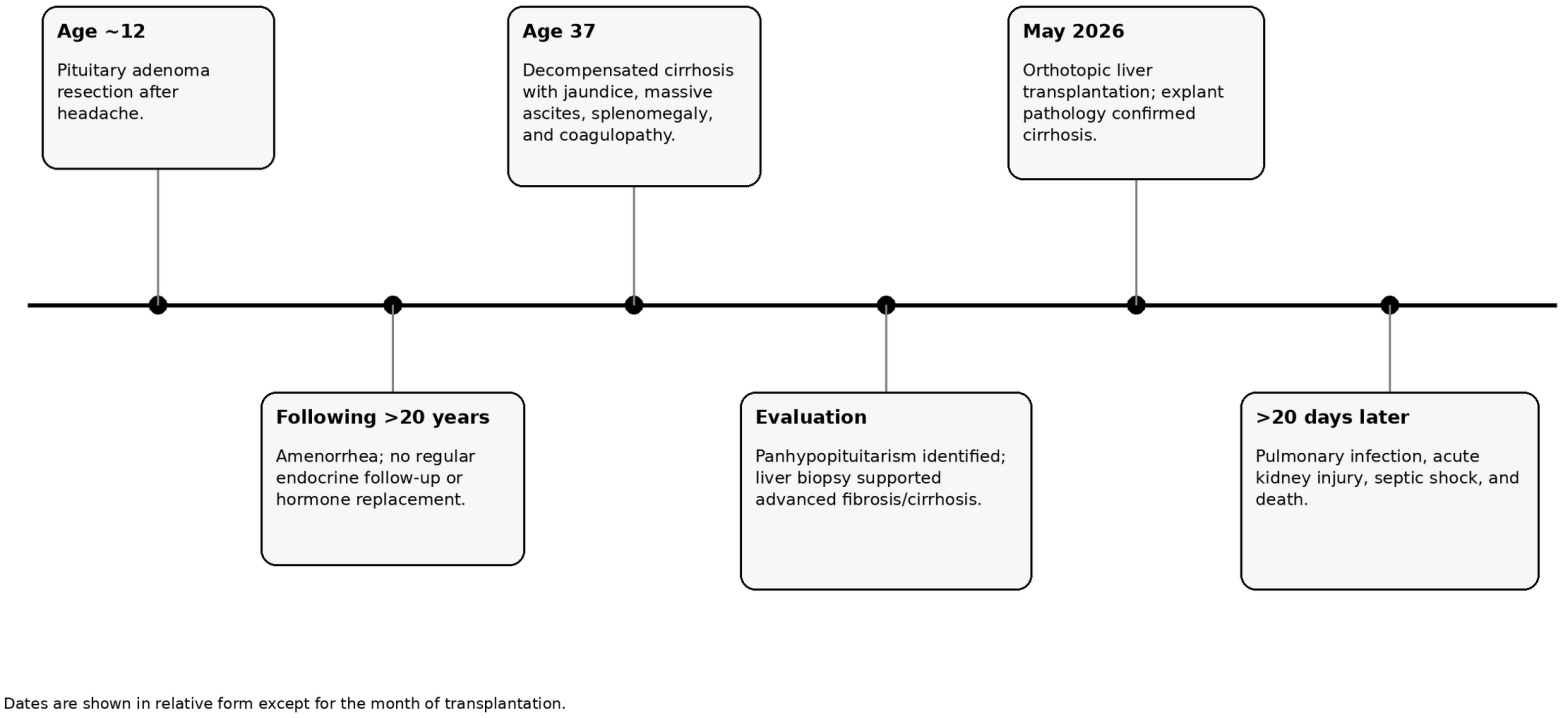
CARE-style clinical timeline. Timeline showing pituitary adenoma surgery in childhood, more than two decades without regular endocrine follow-up or hormone replacement, presentation with decompensated cirrhosis, endocrine and histological evaluation, liver transplantation in May 2026, and death from septic shock more than 20 days later.

At approximately 12 years of age, a pituitary tumor was diagnosed after headache and surgically resected. Amenorrhea followed, with no regular endocrine surveillance or hormone replacement for more than two decades. The original operative record and pathology were unavailable. The clinical timeline is shown in **Figure 1**.

On admission, she had jaundice, massive ascites, splenomegaly, leg edema, and absent axillary and pubic hair. Initial testing showed total bilirubin 156.6 μmol/L, albumin 30.6 g/L, INR 2.64, platelets 33 ×10^9^/L, sodium 123.4 mmol/L, and creatinine 29.2 μmol/L. During pre-transplant evaluation, total bilirubin reached 278.7 μmol/L; by early May, creatinine was 97 μmol/L, PT 20.4 s, prothrombin activity 30.7%, APTT 52.4 s, hemoglobin 69 g/L, and platelets 20 ×10^9^/L. She had Child-Pugh class C disease and an estimated MELD-Na of 30–31 (**Table 1**).

**Table 1:** Key clinical, biochemical, endocrine, imaging, microbiological, histological, and post-transplant findings.

Category	Variable	Result(s) with time point
Background	Age/anthropometrics	At presentation: 37 years; 162 cm; 73.6 kg; BMI 28.04 kg/m²^a^
Liver biochemistry	AST/ALT	Initial: 141/37 U/L; repeat: 84/29.9 U/L
Liver biochemistry	Total/direct bilirubin	Initial: 156.6/13.5 μmol/L; referral evaluation: 278.7/89.6 μmol/L; late pre-transplant: 144.7/53.6 μmol/L
Liver biochemistry	Albumin	Initial: 30.6 g/L; repeat: 26.1 g/L; late pre-transplant: 27.3 g/L
Coagulation	PT/PTA/APTT/INR	Initial: PT 27.7 s, INR 2.64; referral evaluation: PT 19.6 s, PTA 32.4%; late pre-transplant: PT 20.4 s, PTA 30.7%, APTT 52.4 s
Hematology	WBC/hemoglobin/platelets	Referral evaluation: 12.05 ×10^9^/L/122 g/L/51 ×10^9^/L; early May: WBC 2.58 ×10^9^/L, platelets 25 ×10^9^/L; late pre-transplant: hemoglobin 69 g/L, platelets 20 ×10^9^/L
Electrolytes/renal	Sodium/potassium/creatinine	Initial: 123.4 mmol/L/2.97 mmol/L/29.2 μmol/L; repeat: 124.9 mmol/L/2.59 mmol/L/37.3 μmol/L; late pre-transplant creatinine: 97 μmol/L
Severity	Child-Pugh/MELD-Na	At initial admission: class C/approximately 30-31
Iron indices	Ferritin/transferrin saturation	Ferritin 250.9 ng/mL; transferrin saturation 94.6%^d^
Adrenal axis	Pretreatment cortisol/ACTH	00:00: 14.4 μg/dL/1.12 pg/mL; 08:00: 11.91 μg/dL/1.25 pg/mL; 16:00: 8.11 μg/dL/<1 pg/mL^b^
Thyroid axis	TSH/FT4/T3	Referral evaluation: 2.54 mIU/L/9.17 pmol/L/2.75 pmol/L
Gonadal axis	LH/FSH/estradiol	Referral evaluation: <0.1 mIU/mL/0.599 mIU/mL/61.3 pg/mL
Prolactin	Prolactin	Referral evaluation: 4.23 ng/mL
GH/IGF-1 axis	Random GH/IGF-1	Initial GH: 0.44 and 0.97 ng/mL; IGF-1: <15 ng/mL twice; later random GH during liver failure: >50 ng/mL^c^
Posterior pituitary	Water balance	Polyuria; clinical diagnosis of central diabetes insipidus
Copper evaluation	Ceruloplasmin/K-F ring	Ceruloplasmin 161.9 mg/L; K-F ring not observed on examination^d^
Imaging	CT/SWE/PET-CT	Cirrhosis, portal hypertension with esophagogastric varices, SWE 55 kPa, massive ascites, and splenomegaly
Ascitic fluid	Culture	Pre-transplant: Candida tropicalis, susceptible to caspofungin; repeat culture negative after treatment
Histology	Biopsy/explant	Short biopsy supported advanced fibrosis/cirrhosis; explant pathology showed micronodular cirrhosis with hepatocellular cholestasis; gallbladder pathology showed cholelithiasis with chronic cholecystitis^e^
Post-transplant	Early course and outcome	Aminotransferases declined; platelets 18 ×10^9^ /L; pulmonary infection and AKI requiring hemodialysis; septic shock and death >20 days later

Objective findings are presented in the main table; interpretive caveats and selected reference intervals are provided in the footnotes.

^a^ BMI was influenced by massive ascites and edema.

^b^ Persistently low ACTH suggested central HPA-axis dysfunction; however, the morning total cortisol was indeterminate, and secondary adrenal insufficiency was not confirmed by dynamic testing.

^c^ Very low IGF-1 in advanced cirrhosis may reflect impaired hepatic synthesis and GH resistance; adult GH deficiency was therefore not formally established.

^d^ Iron overload and Wilson disease were not completely excluded because HFE testing, MRI iron quantification, tissue iron assessment, 24-hour urinary copper, ATP7B testing, and hepatic copper quantification were unavailable; alpha-1 antitrypsin deficiency was not evaluated.

^e^ The short biopsy cores limited etiological assessment. Explant pathology confirmed micronodular cirrhosis with hepatocellular cholestasis, but this nonspecific pattern did not establish MASLD, MASH, or another specific cause.

Selected adult reference intervals: AST 15–40 U/L, ALT 7–40 U/L, total bilirubin 5–21 μmol/L, and albumin 35–55 g/L. Endocrine reference intervals are assay-, age-, and physiological-state dependent. ACTH, adrenocorticotropic hormone; AKI, acute kidney injury; ALT, alanine aminotransferase; APTT, activated partial thromboplastin time; AST, aspartate aminotransferase; FT4, free thyroxine; FSH, follicle-stimulating hormone; GH, growth hormone; HPA, hypothalamic-pituitary-adrenal; IGF-1, insulin-like growth factor-1; INR, international normalized ratio; K-F, Kayser-Fleischer; LH, luteinizing hormone; MASH, metabolic dysfunction-associated steatohepatitis; MELD-Na, Model for End-stage Liver Disease-Sodium; PT, prothrombin time; PTA, prothrombin activity; SWE, shear-wave elastography.

Before glucocorticoid treatment, cortisol was 14.4 μg/dL at 00:00, 11.91 μg/dL at 08:00, and 8.11 μg/dL at 16:00; the corresponding ACTH concentrations were 1.12, 1.25, and <1 pg/mL. Persistently low ACTH suggested central HPA-axis dysfunction. However, the 08:00 total cortisol was indeterminate, and interpretation was complicated by critical illness, advanced cirrhosis, and possible changes in cortisol-binding proteins and cortisol clearance. Without dynamic testing, secondary adrenal insufficiency could not be confirmed and was considered a probable clinical diagnosis. FT4 was 9.17 pmol/L with TSH 2.54 mIU/L, compatible with central hypothyroidism given the inappropriately normal TSH. LH was <0.1 mIU/mL, FSH 0.599 mIU/mL, estradiol 61.3 pg/mL, and prolactin 4.23 ng/mL. Initial random GH values were 0.44 and 0.97 ng/mL, IGF-1 was <15 ng/mL twice, and a later random GH exceeded 50 ng/mL during severe liver failure. Because random GH is non-diagnostic and cirrhosis lowers hepatic IGF-1 production, adult GH deficiency could not be confirmed; the findings were termed severe GH/IGF-1 axis disturbance. Central diabetes insipidus was diagnosed, and glucocorticoid, levothyroxine, and desmopressin were initiated.

**Figure 2:**
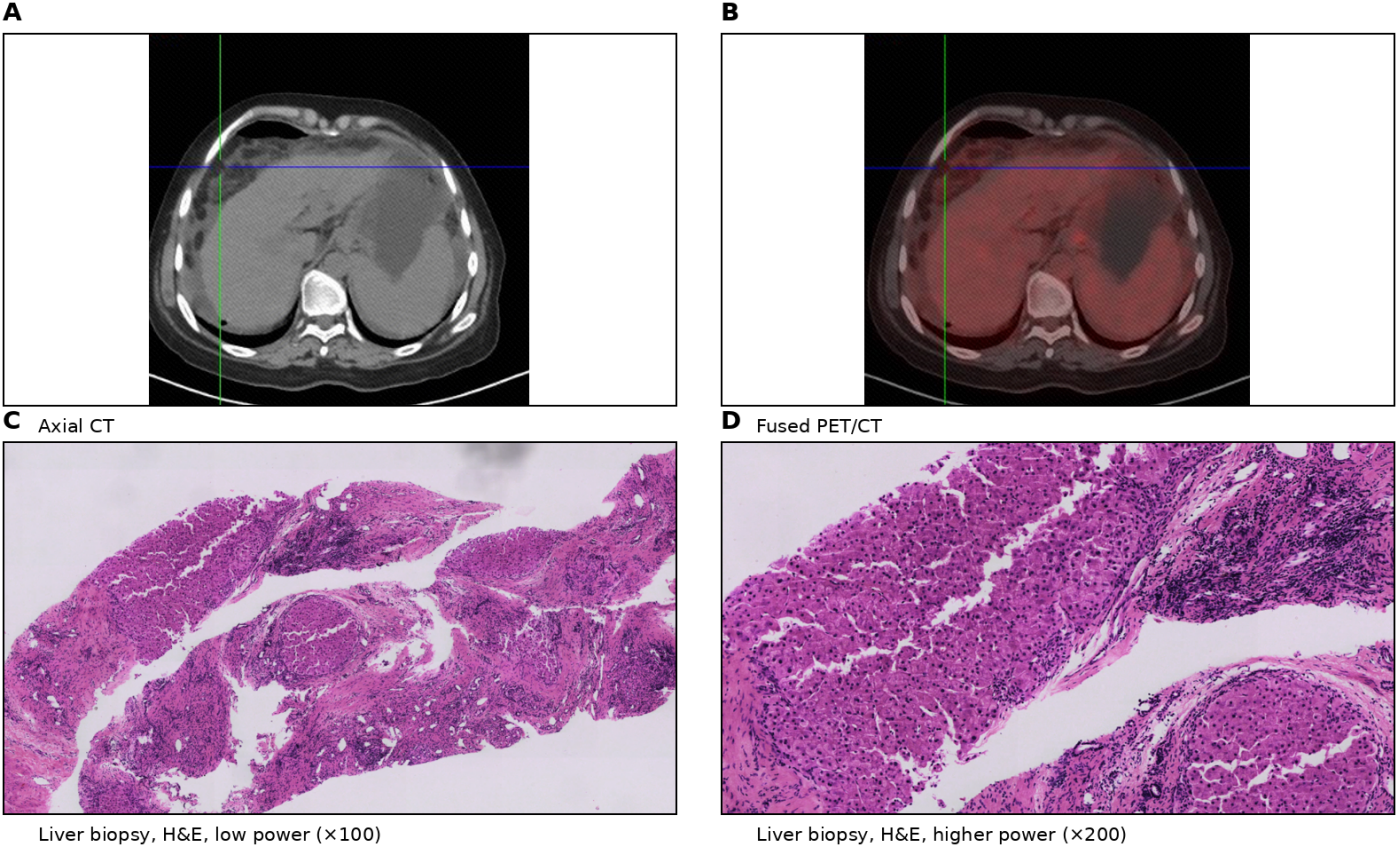
Radiological and histological evidence of advanced chronic liver disease. **(A)** Axial CT image from PET/CT showing massive ascites; reduced liver volume was noted in the radiology report. **(B)** Fused PET/CT image showing massive ascites and marked splenomegaly. **(C)** Hematoxylin and eosin staining of the liver biopsy at original magnification ×100, showing disrupted hepatic architecture with fibrotic expansion. **(D)** Hematoxylin and eosin staining at original magnification ×200, showing hepatocellular vacuolation and architectural distortion. The short biopsy supported advanced fibrosis/cirrhosis but was insufficient for a definitive diagnosis of MASH or reliable assessment of iron or copper deposition.

Ultrasound showed a steatotic liver, ascites, and splenomegaly; shear-wave elastography measured 55 kPa. CT demonstrated cirrhosis, portal hypertension with distal esophageal and gastric varices, marked splenomegaly, and massive ascites. PET-CT showed reduced liver volume, gallstones, reactive marrow uptake, and no obvious recurrent sellar lesion (**Figures 2A, B**). Ascitic-fluid culture grew *Candida tropicalis* susceptible to caspofungin; repeat culture was negative after treatment. Echocardiography showed preserved systolic function, and bone marrow findings were interpreted in the setting of hypersplenism and systemic illness.

Testing did not support common viral, autoimmune, alcohol-related, drug-related, or overt cardiac causes. Ferritin was 250.9 ng/mL and transferrin saturation 94.6%. Although reduced transferrin synthesis in advanced cirrhosis can increase transferrin saturation (15), *HFE* testing, MRI iron quantification, and tissue iron assessment were unavailable; iron overload remained unexcluded. Ceruloplasmin was 161.9 mg/L, and no Kayser-Fleischer ring was observed. However, 24-hour urinary copper, *ATP7B* testing, and hepatic copper quantification were not performed, so Wilson disease was not formally excluded (16). Alpha-1 antitrypsin deficiency was also not evaluated.

Liver biopsy yielded short tissue strips measuring 0.3–0.6 cm. Histology showed disrupted lobular architecture, hepatocellular vacuolation, and collagen proliferation (**Figures 2C, D**). The specimen supported advanced fibrosis/cirrhosis but was insufficient to establish or exclude metabolic dysfunction-associated steatohepatitis or to assess iron and copper deposition reliably. In May 2026, she underwent orthotopic liver transplantation for acute-on-chronic liver failure. Explant pathology showed micronodular cirrhosis with hepatocellular cholestasis; the gallbladder showed cholelithiasis with chronic cholecystitis. Micronodular cirrhosis is a nonspecific architectural pattern and did not establish MASLD, MASH, or another specific etiology. Aminotransferase levels declined postoperatively, but severe thrombocytopenia persisted, and pulmonary infection and acute kidney injury requiring hemodialysis developed. Despite intensive supportive treatment, she progressed to septic shock and died more than 20 days after transplantation.

## Discussion

Her presentation was consistent with long-standing post-surgical panhypopituitarism, although the certainty of each hormonal deficit differed. Pretreatment ACTH was persistently low, but the 08:00 total cortisol of 11.91 μg/dL was indeterminate and dynamic testing was not performed. Critical illness and advanced liver disease can alter ACTH secretion, cortisol-binding proteins, and cortisol clearance; secondary adrenal insufficiency therefore remained a probable clinical diagnosis rather than a confirmed biochemical diagnosis. Markedly reduced IGF-1 also did not establish adult GH deficiency because cirrhosis reduces hepatic IGF-1 synthesis and may cause GH resistance. The discordant random GH values further limited interpretation. Despite these axis-specific uncertainties, the overall clinical and biochemical picture—including postoperative amenorrhea, central hypothyroidism, markedly suppressed gonadotropins, low prolactin, central diabetes insipidus, and severe GH/IGF-1 axis disturbance—strongly supported long-standing panhypopituitarism.

Previously reported severe hepatic complications of hypopituitarism have mainly involved childhood-onset craniopharyngioma or other hypothalamic-pituitary disorders. In several pediatric cases, steatohepatitis was histologically documented, and recurrent graft steatosis or steatohepatitis after liver transplantation—particularly when GH deficiency remained untreated—strengthened the plausibility of an endocrine-metabolic pathway (9, 10, 14). However, alternative or additional contributors were common, including craniopharyngioma-related hypothalamic obesity, diabetes mellitus, interrupted hormone replacement, and, in one report, an inactivating KCNJ11 variant (8, 11, 13, 14). The extent to which viral, autoimmune, genetic, and other metabolic liver diseases were evaluated or excluded was not uniform across the published cases (8–14). Accordingly, the available literature supports a biologically plausible association but not a uniform or exclusive causal pathway. Unlike several earlier reports, our case lacked pre-cirrhotic histology and complete etiological testing, so the pathway from endocrine disease to cirrhosis could not be reconstructed.

Endocrine disorders are increasingly recognized as contributors to MASLD through adverse effects on body composition, insulin sensitivity, and hepatic lipid metabolism (2–7). Disturbance of the GH/IGF-1 axis can promote visceral adiposity, altered hepatic lipid handling, insulin resistance, oxidative stress, inflammation, and fibrosis (2–4). Central hypothyroidism and hypogonadotropic hypogonadism may add adverse metabolic effects. Nevertheless, progression to decompensated cirrhosis or end-stage liver disease remains uncommon (8–14). Taken together, this case supports a biologically plausible association between long-standing untreated panhypopituitarism and advanced chronic liver disease, but it does not establish a causal relationship. This distinction is particularly important because decompensated cirrhosis itself alters hormone synthesis, binding proteins, and endocrine test results.

The main limitation was the incomplete etiological evaluation of cirrhosis. Transferrin saturation of 94.6% required further assessment, although low transferrin in advanced cirrhosis may produce a misleading elevation (15). The modestly low ceruloplasmin and absence of an observed Kayser-Fleischer ring did not exclude Wilson disease because urinary copper, ATP7B testing, and hepatic copper quantification were unavailable. Hemochromatosis, Wilson disease, alpha-1 antitrypsin deficiency, and other metabolic disorders were therefore not definitively excluded. The biopsy cores measured only 0.3–0.6 cm, which restricted reliable etiological assessment of steatohepatitis, iron or copper deposition, and other informative histological features. The explant showed micronodular cirrhosis with hepatocellular cholestasis; however, micronodular cirrhosis is nonspecific and is not diagnostic of MASLD or MASH. These findings confirmed advanced chronic liver disease but did not establish its etiology, and the available pathology assessment lacked the detail needed to evaluate iron, copper, or alpha-1 antitrypsin deposition reliably. In this patient, untreated panhypopituitarism remains a possible co-factor rather than a confirmed or sole cause of cirrhosis.

## Patient perspective

A patient perspective could not be obtained because the patient died during the post-transplant course.

## Conclusion

We report decompensated cirrhosis requiring transplantation in a woman with more than two decades of untreated panhypopituitarism after pituitary surgery. The case supports a biologically plausible association between long-standing untreated panhypopituitarism and advanced chronic liver disease; however, a direct causal relationship cannot be inferred from a single case, particularly because iron overload, Wilson disease, alpha-1 antitrypsin deficiency, and histological steatohepatitis were not fully excluded. Lifelong endocrine follow-up after pituitary surgery should include attention to metabolic and hepatic complications.

## Data Availability

The original contributions presented in the study are included in the article/supplementary material. Further inquiries can be directed to the corresponding authors.
